# Supplementary material for: A meta-analysis of immune-cell fractions at high resolution reveals novel associations with common phenotypes and health outcomes
Source: Genome Med. 2023 Jul 31;15:59. doi: 10.1186/s13073-023-01211-5 (PMC10388560; doi:10.1186/s13073-023-01211-5)
Supplement: Supplementary file 2 — Additional file 2. A pdf Supplementary Information file. Contains all Supplementary. [file 13073_2023_1211_MOESM2_ESM.pdf]

SUPPLEMENTARY INFORMATION

This document contains all Supplementary Figures and their legends.

Supplementary Figures

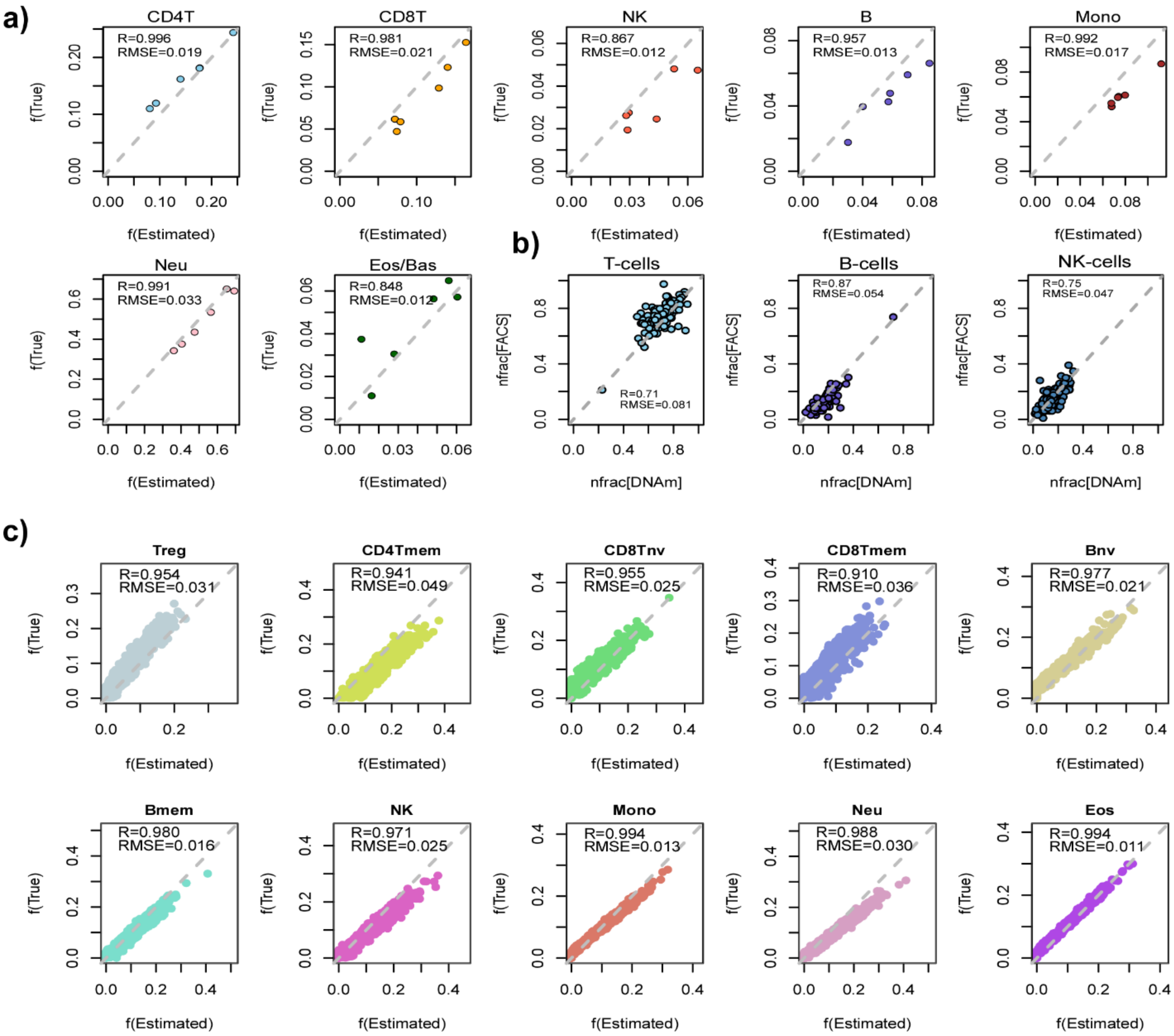

fig.S1: Validation of the EPIC 12 immune cell-type reference DNAm matrix in Illumina 850k DNAm data with matched FACS counts and in *in-silico* WGBS mixtures. a) Scatterplots of true fractions vs estimated fractions for 6 whole blood EPIC DNAm profiles with matched FACS cell-counts for 7 cell-types, as shown. Here f(True) denotes the cell-fraction estimate from FACS. For each estimated cell-type we display the R-value (Pearson Correlation Coefficient) and root mean square error (RMSE). **b)** As a), but now for 144 peripheral blood EPIC DNAm profiles with matched FACS counts for 3 immune cell-types. The x and y-axis display the normalized fractions from DNAm and FACS, respectively, normalized to these 3 cell-types. **c)** For each of 10 immune cell types, scatterplots of true mixture weights (y-axis) vs estimated fractions (x-axis) for 1000 in-silico mixtures of WGBS DNAm profiles from IHEC. Each mixture is a linear combination of 10 immune cell sorted samples with weights drawn randomly from a uniform distribution. Pearson R-value and RMSE are given.

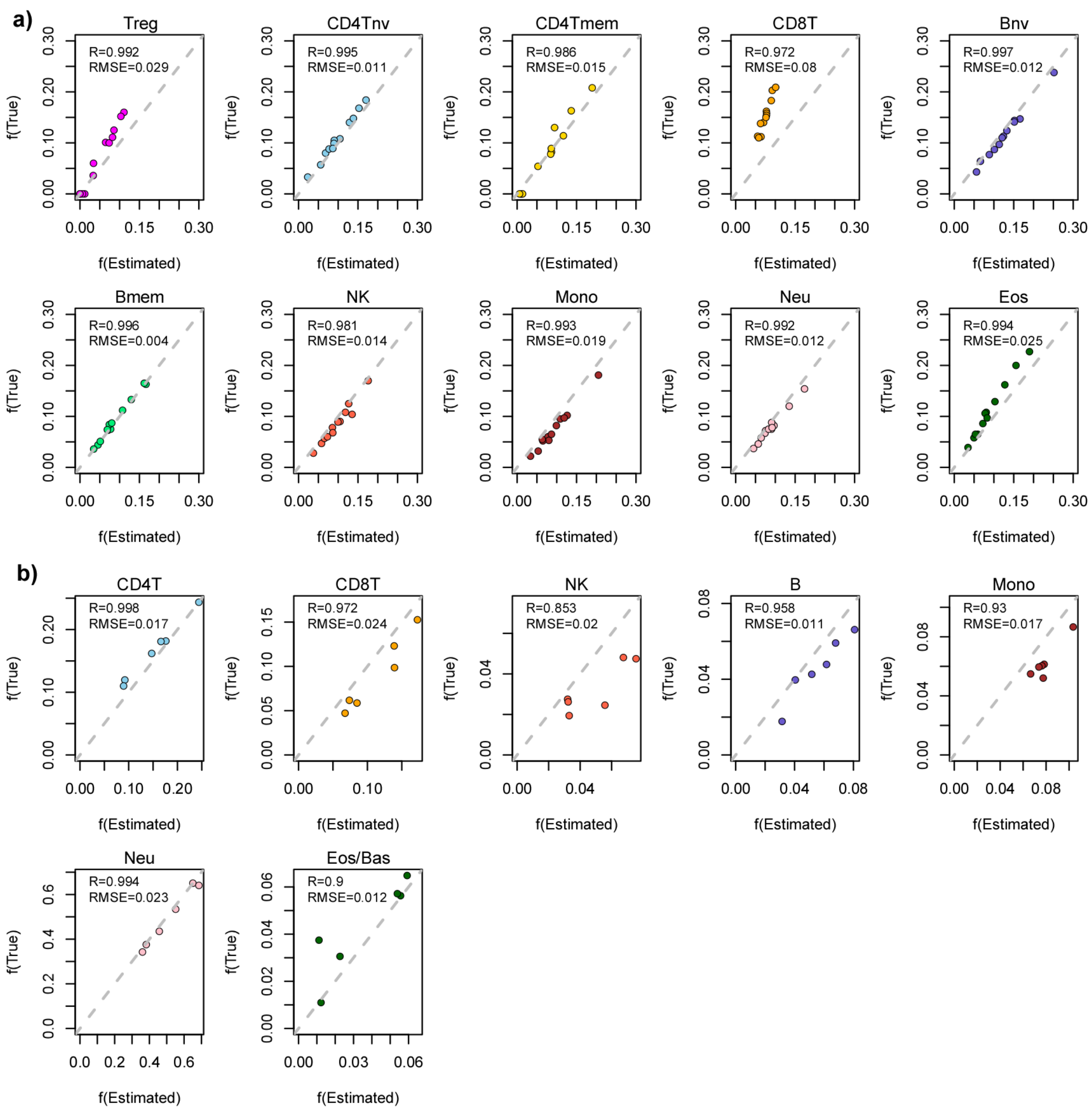

fig.S2: Validation of the 450k 12 immune cell-type reference DNAm matrix. a) Scatterplots of true fractions vs estimated fractions for 10 blood cell subtypes using the EPIC DNAm data from 10 artificial mixtures where the underlying mixing proportions were known. The cell-type fractions were estimated using the 12 blood cell-type reference DNAm matrix restricted to 450k probes. For each estimated cell-type we display the R-value (Pearson Correlation Coefficient) and root mean square error (RMSE). **b)** As a), but now for 6 whole blood EPIC DNAm profiles with matched FACS cell-counts for 7 cell-types, as shown. Here f(True) denotes the cell-fraction from FACS.

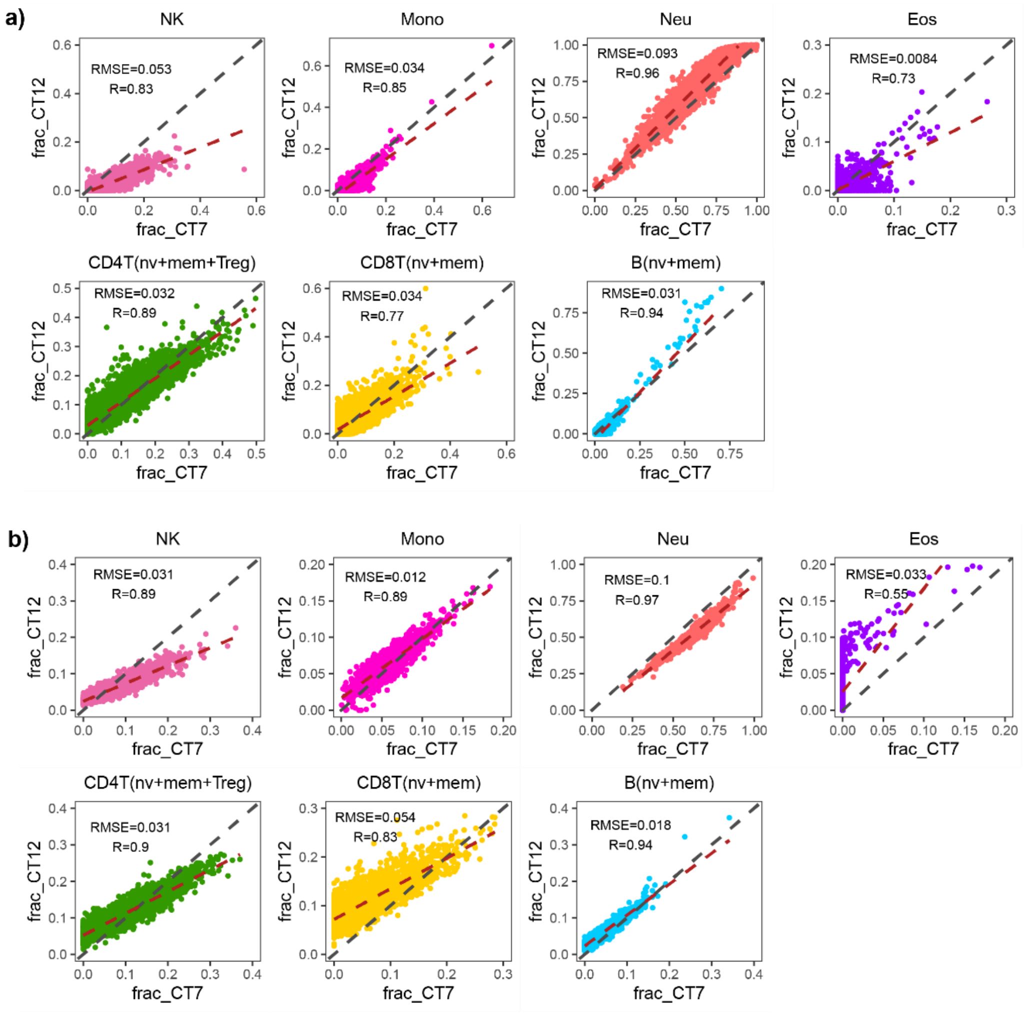

fig.S3: Comparison of estimated fractions between the 12 and 7 immune-cell type DNAm reference matrices. a) Scatterplots of 7 pooled immune-cell fractions estimated with our 12 cell-type DNAm reference matrix (y-axis) against the corresponding immune-cell fractions derived with our previous 7 immune-cell DNAm reference matrix, as obtained in the TD7k EPIC DNAm dataset. Grey dashed line indicates y=x, red dashed line is the fitted linear regression. **b)** As **a)**, but for the Lehne 450k DNAm dataset.

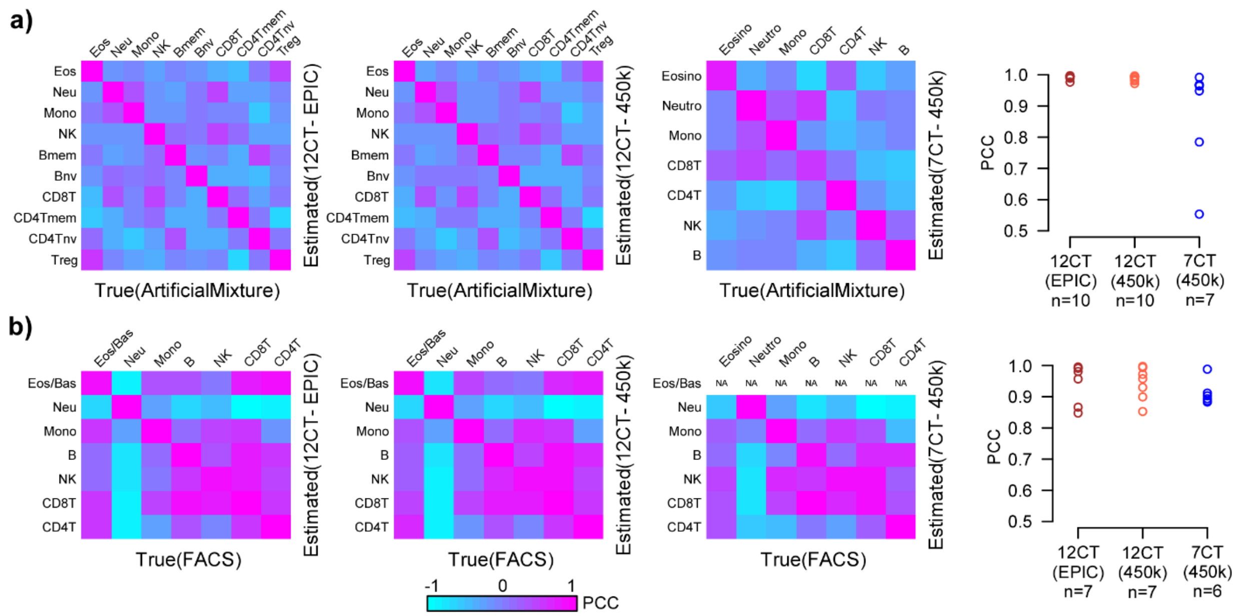

fig.S4: Correlation of estimated fractions from the 12 and 7 immune-cell type DNAm reference matrices with known and measured experimental cell-counts. a) Left three panels depict confusion Pearson Correlation Coefficient (PCC) matrices between estimated immune cell fractions (y-axis) and true fractions (x-axis), as derived by applying the 12 immune-cell type DNAm reference matrix (EPIC) (left), the 12 immune-cell type DNAm reference matrix restricted to 450k probes (middle) and a 7 immune-cell type DNAm reference matrix (right), to 12 artificial mixtures of 10 immune cell-types. Rightmost panel depicts the actual PCC-values (diagonal entries of the confusion matrices) for each of the three DNAm reference matrices. **b)** As a), but applied to to 6 whole blood samples with matched flow cytometric counts for 7 immune cell types.

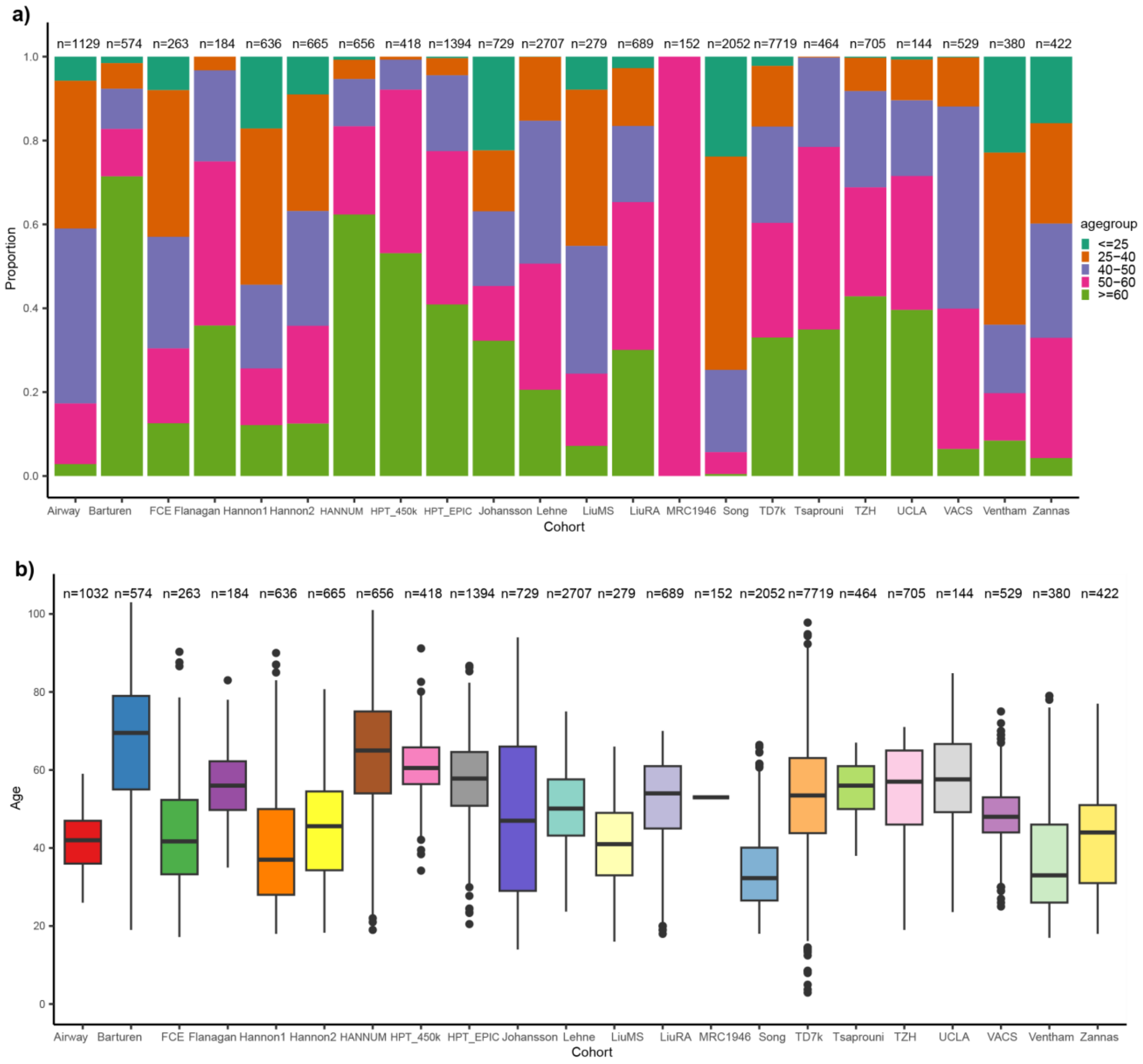

fig.S5: Age-distribution of samples for the 22 cohorts. a) Barplots displaying for each of the 22 datasets, the proportion of samples falling in one of five age-bins, as shown. The total number of samples in each study is displayed at the top of the bars. **b)** Boxplots displaying the full range of ages in each of the 22 cohorts.

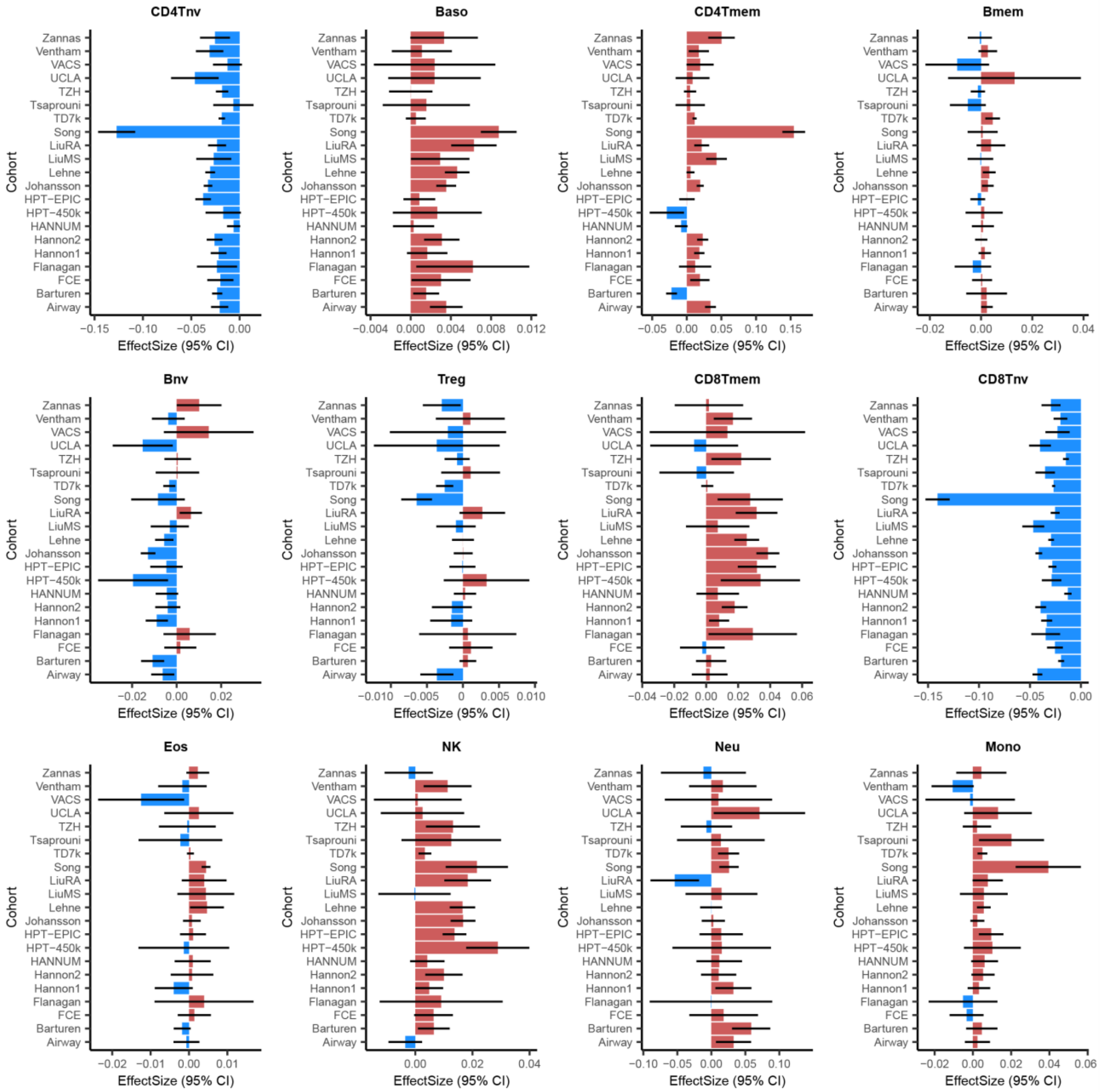

fig.S6: Forest plots of effect size estimates for age for all immune cell fractions and cohorts. For each of the 12 immune-cell types, we display a forest plot of estimated effect sizes (plus their 95% confidence intervals) for age (positive effect sizes means higher fraction in older people) across the 21 cohorts. Estimates have been multiplied by 50 to reflect the percentage change over a 50-year period.

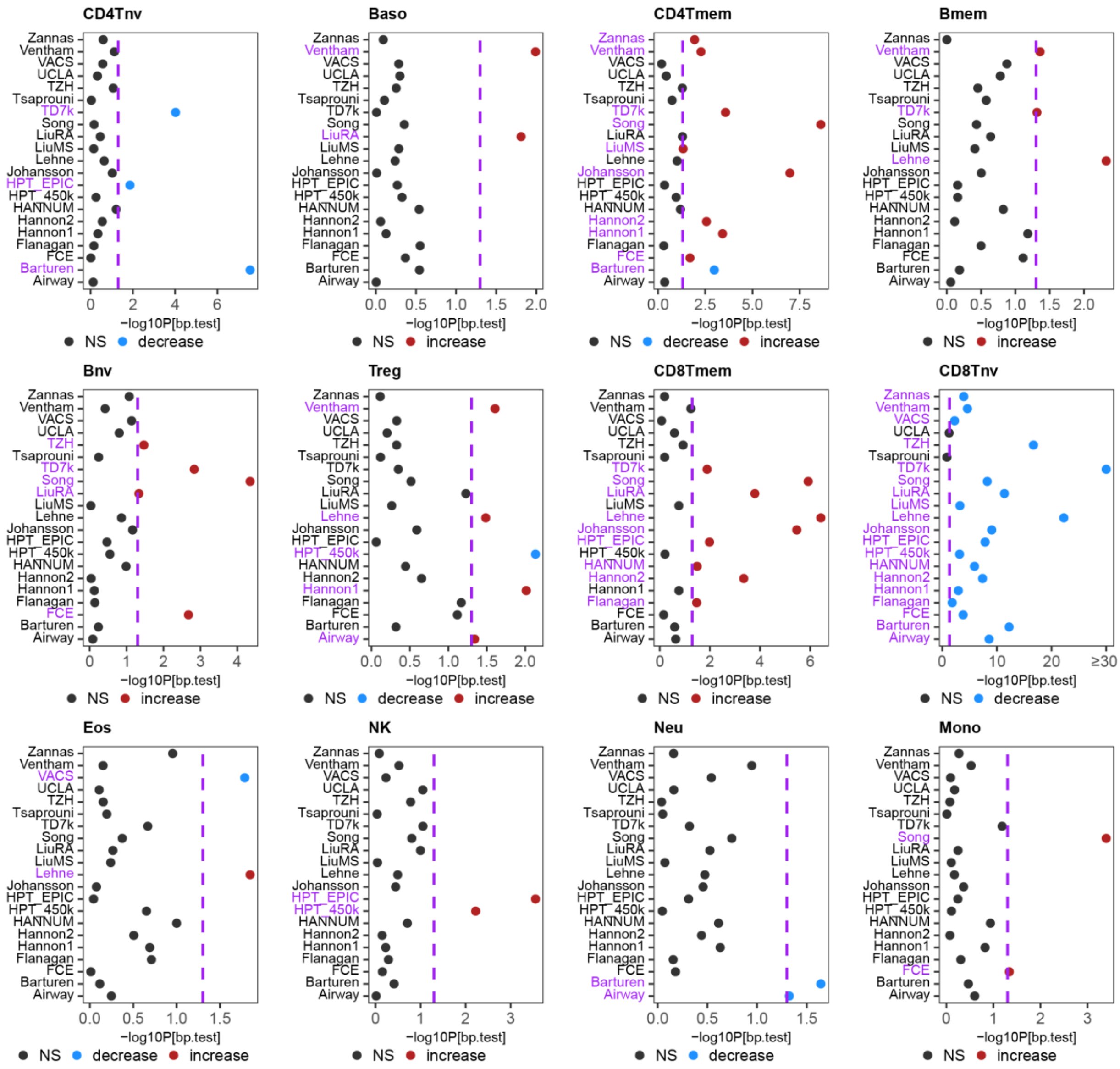

fig.S7: Analysis of heteroscedasticity of immune cell fractions with age. Scatterplots of -$\log_{10}$P-values from the Breusch-Pagan test (x-axis) assessing heteroscedasticity of immune cell-fractions with chronological age. Each panel is one for one immune cell subtype, and y-axis labels the individual cohorts. Vertical dashed line is the line P=0.05. Associations describing increased variance of the cell-type fraction with age are displayed in brown, those describing decreased variance in blue, with non-significant associations shown in black.

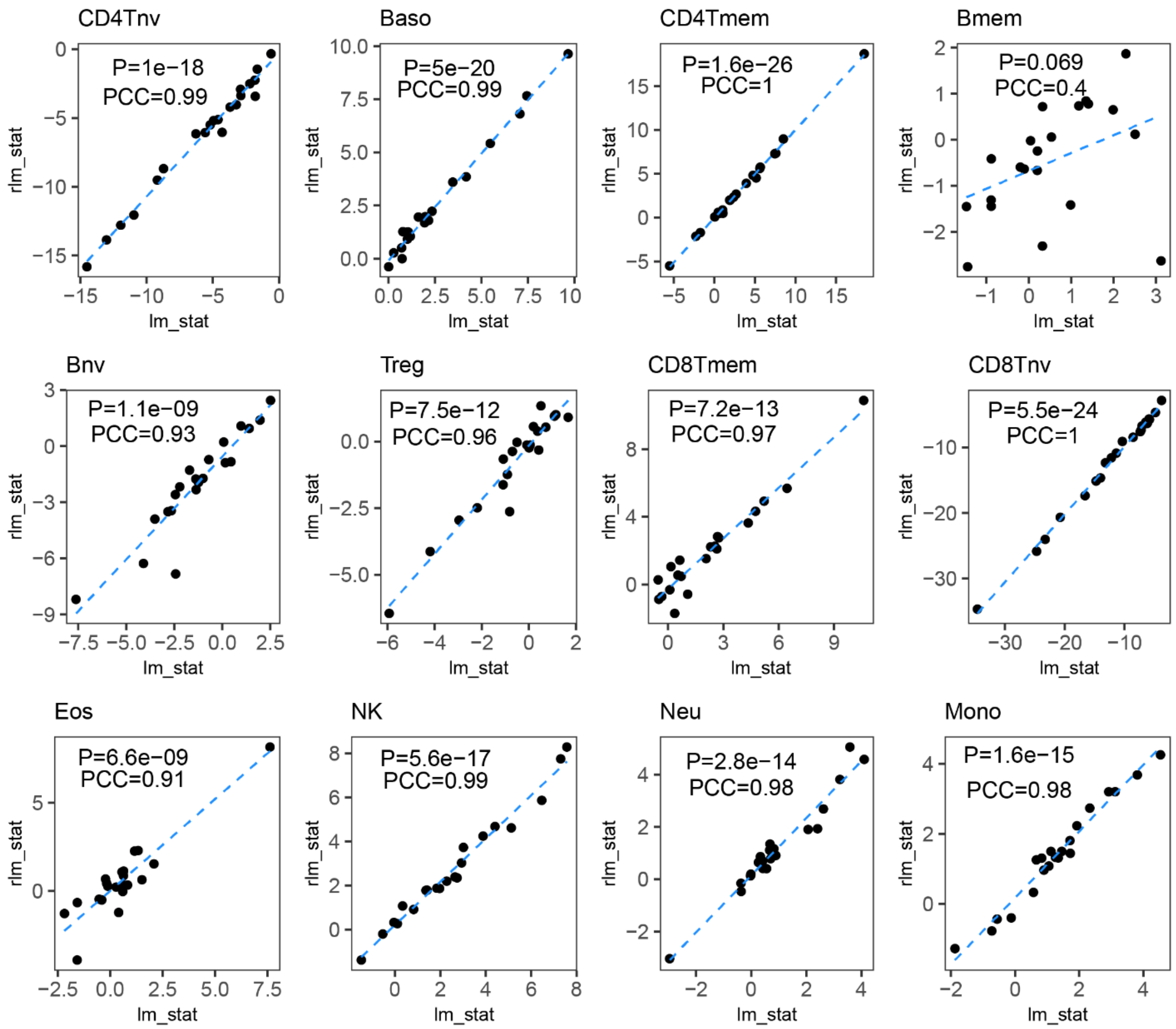

fig.S8: Congruence between robust and ordinary linear regression. Scatterplot of t-statistics from a robust linear model regression (y-axis) of immune-cell fraction against chronological age, versus the corresponding t-statistics derived from an ordinary linear regression model (x-axis), for each immune cell-type and with each datapoint representing one of 21 different cohorts. Both robust and ordinary linear models, the regressions were multivariate adjusting for additional confounders, which was cohort dependent. In each panel, we give the Pearson Correlation Coefficient and associated P-value.

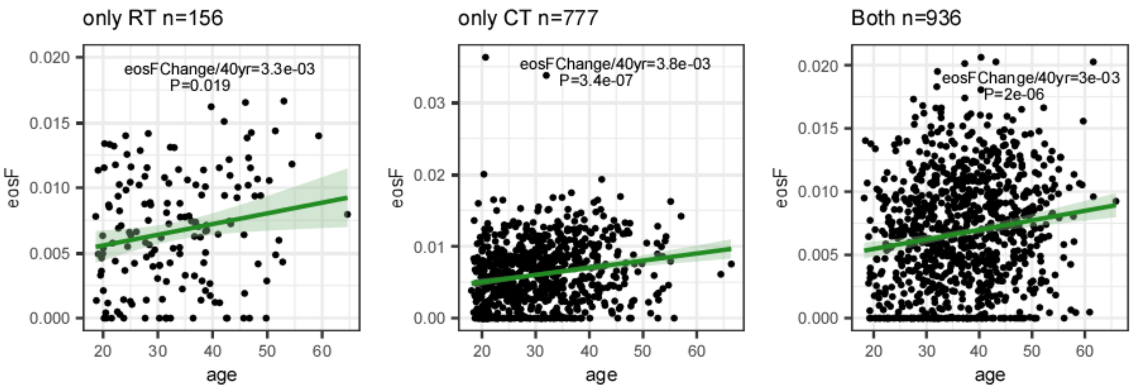

fig.S9: Associations of eosinophil fraction with age in Song et al dataset. Scatterplots of eosinophil fraction (eosF, y-axis) vs age (x-axis) for blood samples from childhood cancer survivors, stratified by type of treatment: left panel displays patients who only received radiotherapy (RT), middle panel displays patients that only received chemotherapy (CT), right panel displays patients who received both RT and CT. Number of samples in each strata is given above plot. Within the plots we provided the change in eosinophil fraction across a 40-year age gap, as well as the associated P-value from a linear regression.

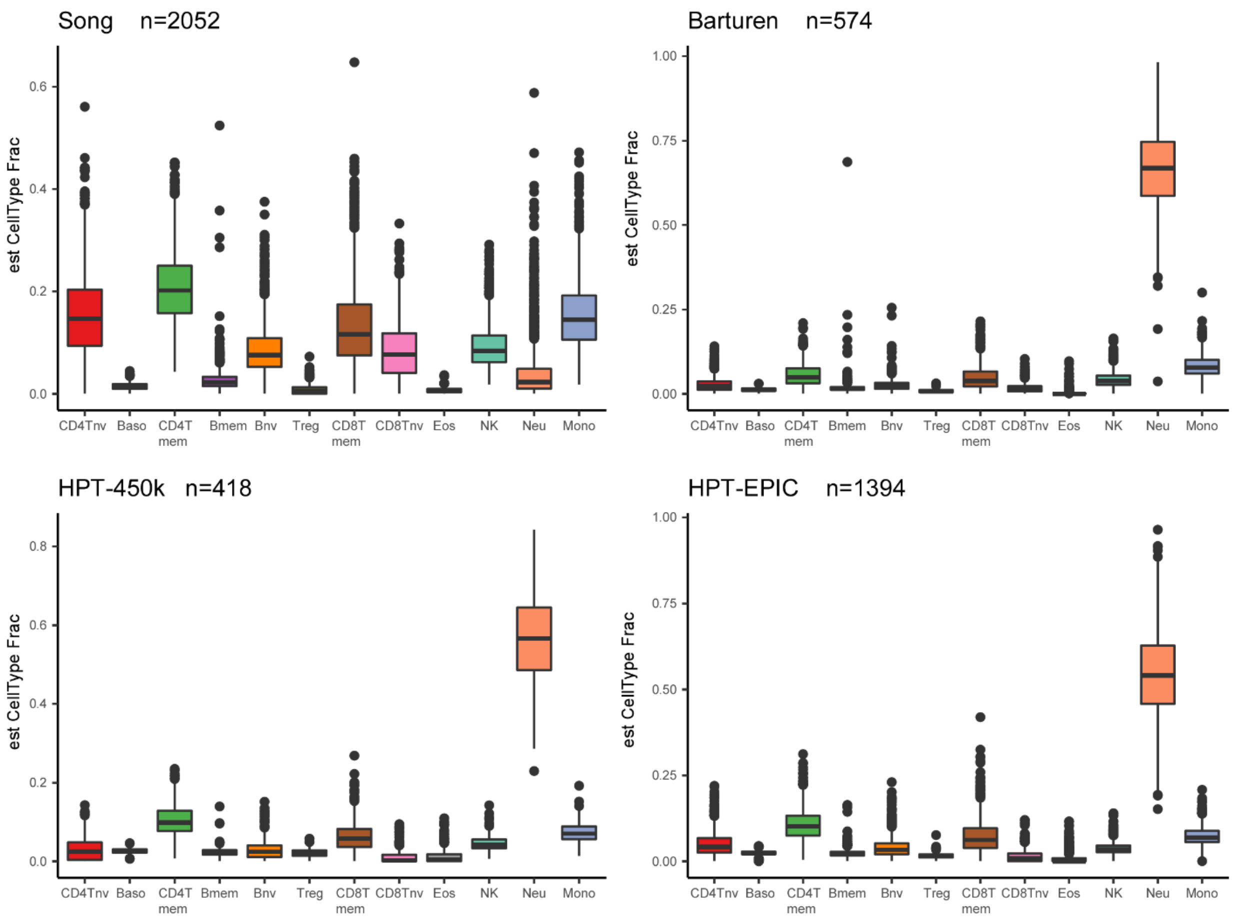

fig.S10: Comparison of estimated cell-type fractions between Song and other large cohorts. Boxplots of estimated blood cell-type fractions for Song et al and 3 other large cohorts.

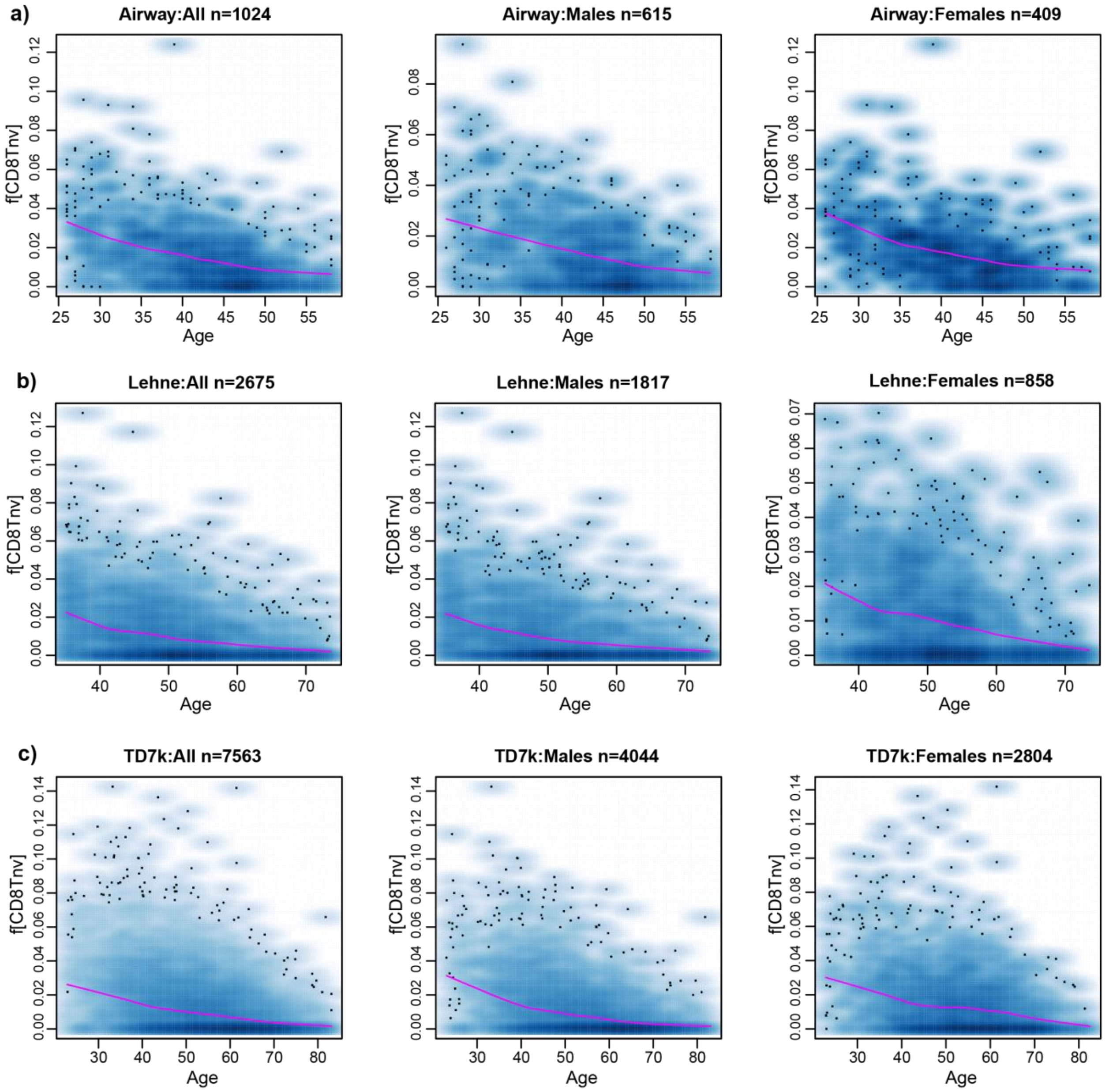

fig.S11: Loess regressions of naïve CD8+ T-cell fraction with age in 3 of the largest cohorts. For each of 3 of the largest cohorts with balanced sample representation across age-groups, we display scatterplots of the naïve CD8+ T-cell fraction against chronological age, with the loess regression curve displayed in magenta. Due to large sample size the 95% confidence intervals are generally too tight to be visible except at some of the extremes in the age distribution.

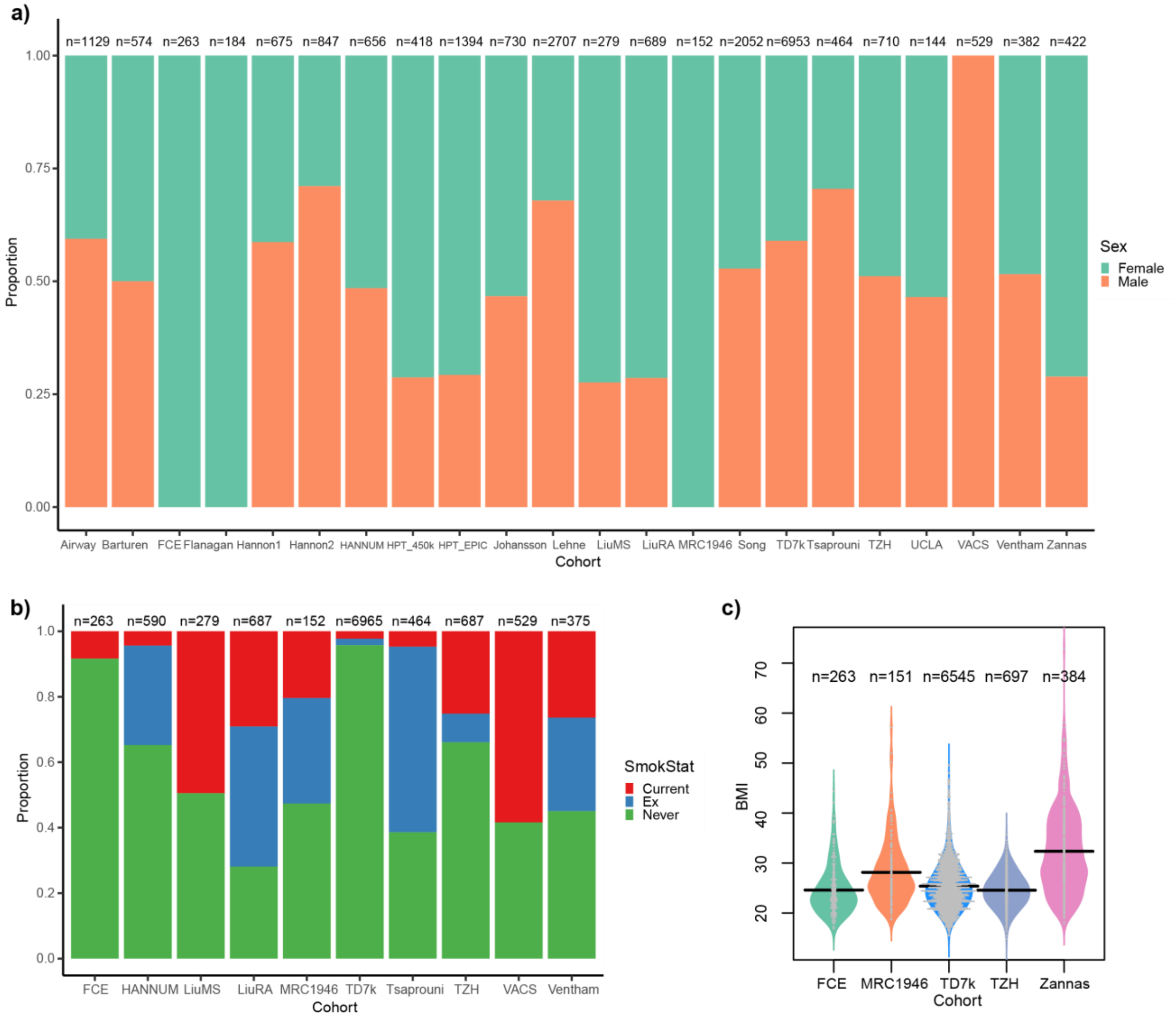

fig.S12: Sex, Smoking and BMI distribution of samples. a) Barplots displaying for each of the 22 datasets, the proportion of males and females. The total number of samples in each study is displayed at the top of the bars. **b)** Barplots displaying for each of 10 datasets with smoking information available, the smoking status of the subjects at sample draw. The total number of samples in each study is displayed at the top of the bars. **b)** Violin plots depicting the distribution of Body Mass Index (BMI) values in the 5 cohorts where BMI was available. Number of samples in each cohort is given.

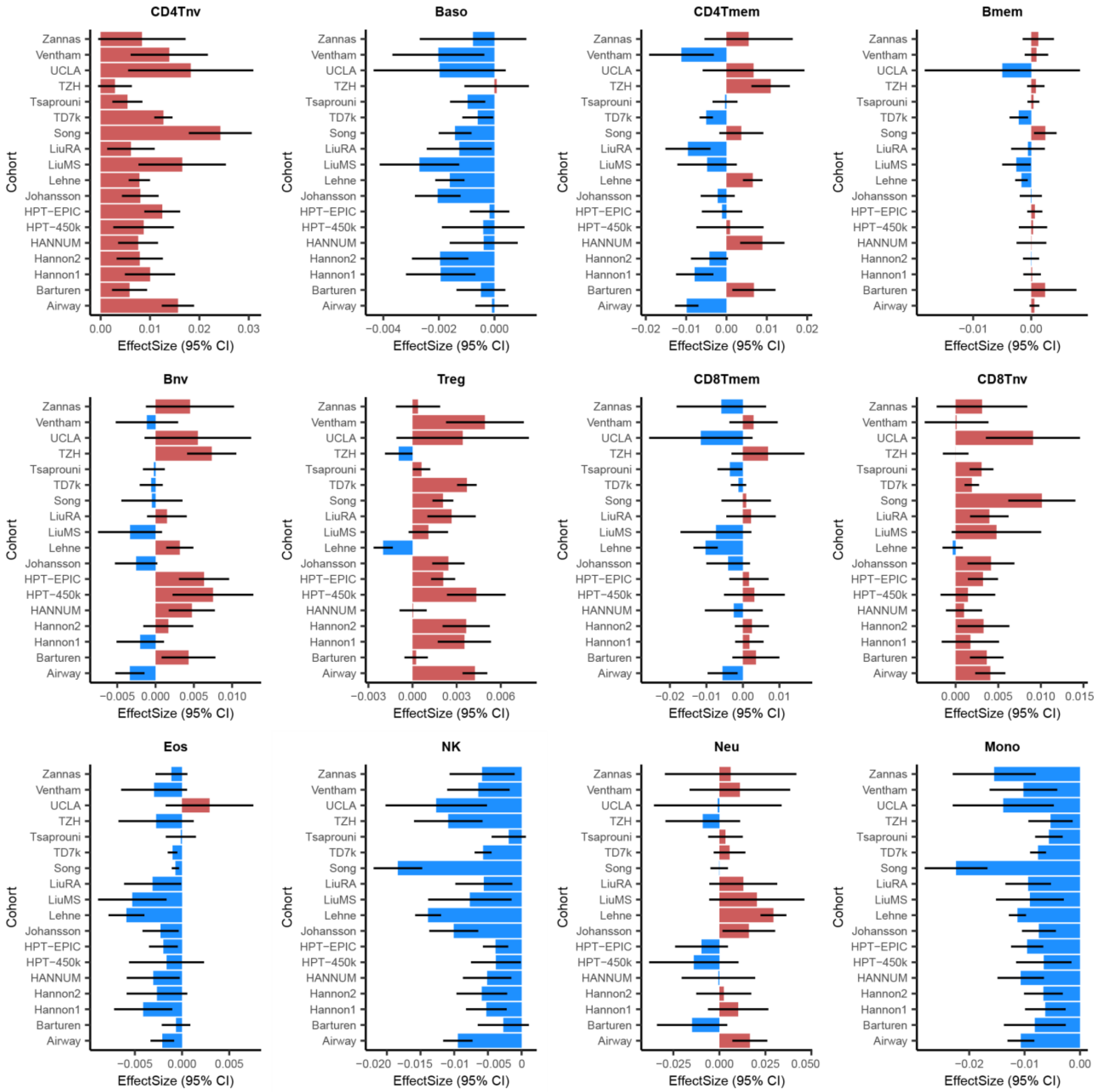

fig.S13: Forest plots of effect size for sex. For each of the 12 immune-cell types, we display a forest plot of estimated effect sizes (plus their 95% confidence intervals) for sex (positive effect sizes means higher fraction in females compared to males) across the 18 cohorts.

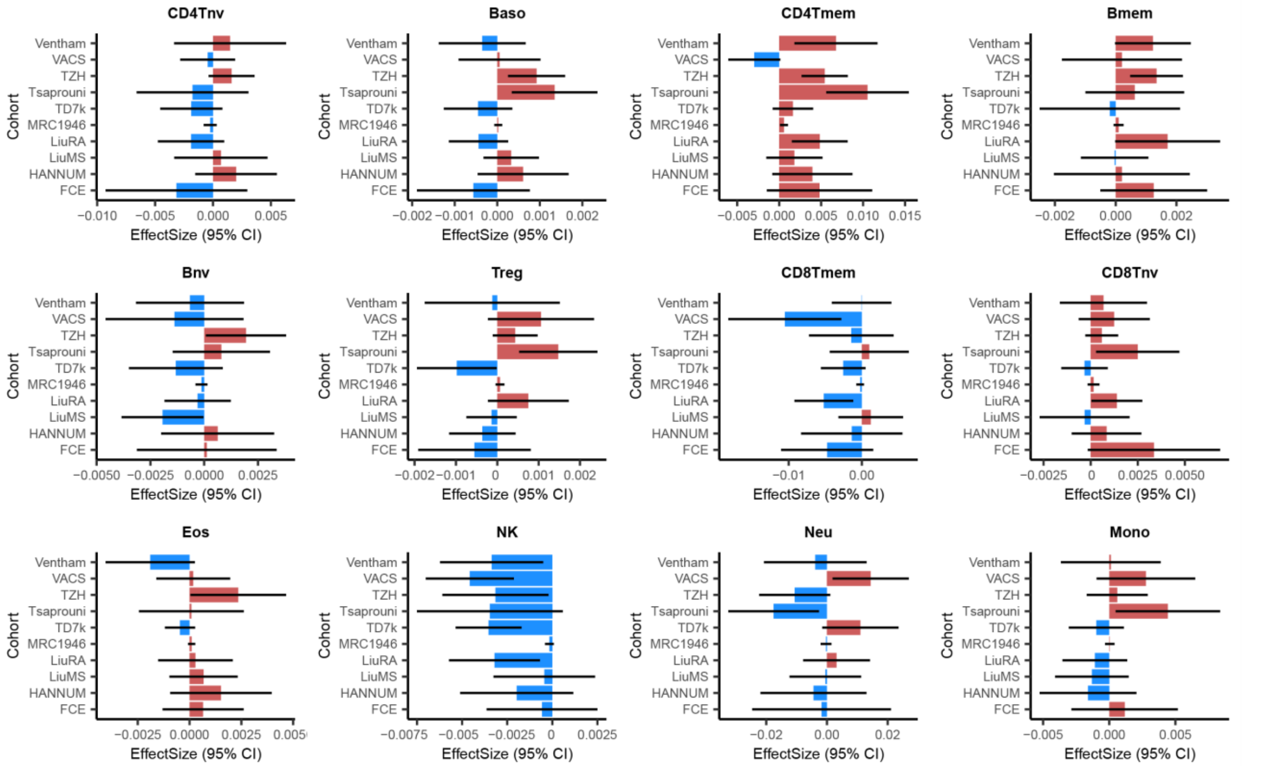

fig.S14: Forest plots of effect size for smoking. For each of the 12 immune-cell types, we display a forest plot of estimated effect sizes (plus their 95% confidence intervals) for smoking across 10 distinct cohorts. Smoking was categorized as 0=never-smoker, 1=ex-smoker, 2=smoker.

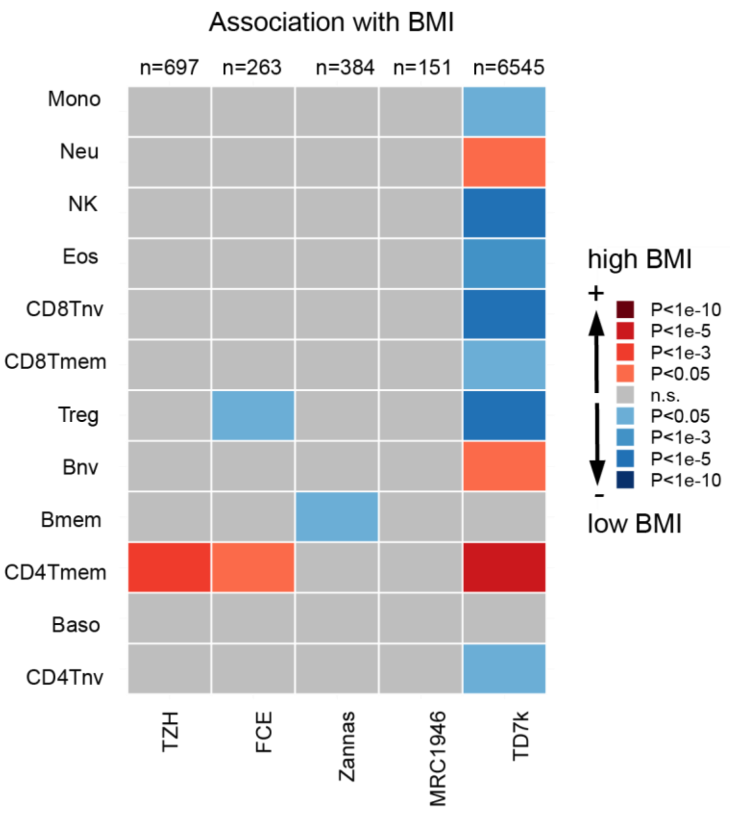

fig.S15: Associations of cell-type fractions with Body Mass Index (BMI). Heatmap displaying the statistical significance and directionality of associations of blood cell-type fractions with BMI for five different cohorts. P-values estimated using multivariate linear regression that included age, sex and smoking status. The number of samples in each cohort is given at the top of the heatmap.

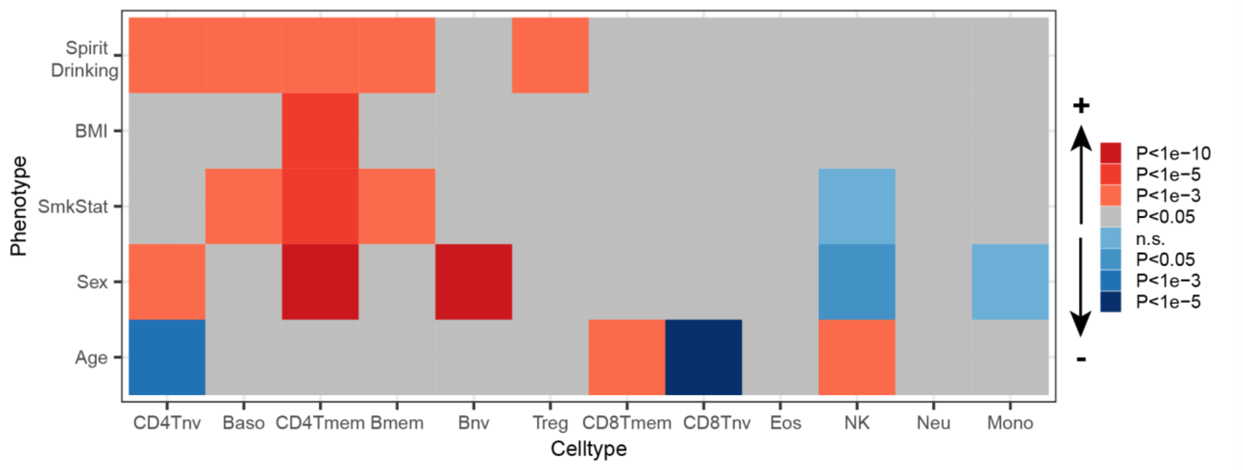

fig.S16: Association of spirit drinking with cell-type fractions in TZH cohort. Heatmap displaying the statistical significance and directionality of associations of blood cell-type fractions with spirit drinking, BMI, smoking status, sex and age. P-values estimated using multivariate linear regression with all 5 covariates.

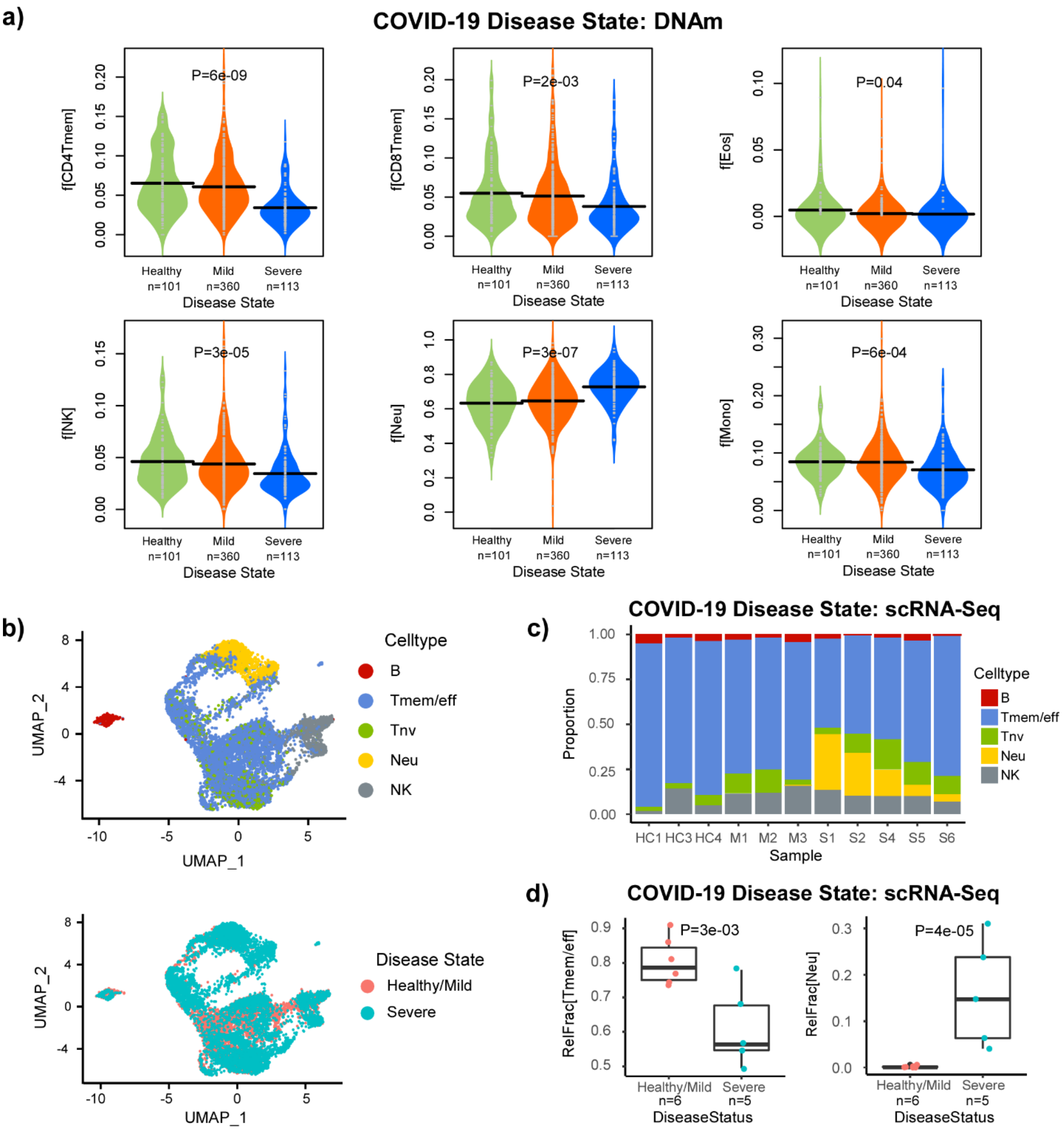

fig.S17: Associations of immune cell-type fractions with Covid-19 severity. a) Violin plots of cell-type fractions (y-axis) against Covid-19 disease severity (x-axis) for all those immune cell-types for which an association was statistically significant. The number of samples in each disease group is given below x-axis. P-value is two-tailed and from a linear regression of cell-type fraction against disease group labeled as 0 (control/negative), 1 (mild) and 2 (severe). **b)** UMAP plots of the scRNA-Seq data of Liao et al profiling immune cells from Covid-19 patients and controls. **c)** Barplot displaying the distribution of immune cell subtype fractions for each of the samples, as inferred from the scRNA-Seq data: HC=healthy control, M=Covid-19 case experiencing mild disease, S=Covid-19 case experiencing severe disease. **d)** Boxplots of immune-cell fraction

against disease state for the two immune cell-types that displayed significant associations with Covid-19 disease state, as inferred from scRNA-Seq data. P-values derive from Propeller.
